# A splicing-derived microRNA from amelogenin exon4 regulates enamel formation via control of exon4 splicing and amelogenin expression

**DOI:** 10.1038/s41598-026-40706-0

**Published:** 2026-02-25

**Authors:** Rozana Shemirani, Trang Duong, Rebecca Kim, Ankitha Shetty, K. Mark Ansel, Yukiko Nakano

**Affiliations:** 1 Department of Orofacial Science, School of Dentistry and Oral and Craniofacial Science Program, Graduate Education and Postdoctoral Affairs, University of California San Francisco, San Francisco, CA USA; 2 Department of Microbiology and Immunology, School of Medicine, University of California San Francisco, San Francisco, CA USA; 3 Center for Oral Health Research, School of Dentistry, University of California San Francisco, San Francisco, CA USA; 4 Department of Orofacial Sciences, School of Dentistry, University of California San Francisco, 513 Parnassus Ave, San Francisco, 94143 CA USA

**Keywords:** Ameloblasts, Dental enamel, Alternative splicing, RNA splicing factors, Amelogenesis, microRNAs, miRNAs, RNA splicing, Enamel, Gene regulation, Reverse transcription polymerase chain reaction, Mechanisms of disease, Amelogenesis imperfecta

## Abstract

Amelogenin, essential for enamel formation, is encoded by the alternatively spliced gene *Amelx*/*AMELX*, with exon4 splicing producing major protein isoforms and a microRNA (miR-exon4). We previously reported that miR-exon4 regulates osteoblast and ameloblast function via the *Nfia*- and *Prkch*-*Runx2* axis and may be involved in X-linked Amelogenesis Imperfecta associated with exon4 splicing defects. This study investigates miR-exon4’s role in enamel defect development and exon4 splicing. A miR-exon4 mimic or inhibitor was applied to mouse pups during enamel development. One-week inhibition of miR-exon4 reduced enamel mineralization, shortened the early mineralization phase, and lowered RUNX2 expression, leading to increased *Amelx* mRNA and amelogenin protein levels. A 24-hour inhibition enhanced exon4 splicing without affecting overall *Amelx* expression, suggesting a direct role in splicing regulation. MiR-exon4 inhibition also altered the expression of Serine/Arginine-rich splicing factors (SRSFs), indicating indirect modulation of exon4 splicing. Nuclear localization of miR-exon4 was confirmed using an *Amelx* minigene, and a mutant form that reduced nuclear miR-exon4 levels impaired its binding to *Amelx* pre-mRNA. A binding site of miR-exon4 at the intron4 branch point further supports its direct involvement in exon4 splicing. These findings suggest a role for miR-exon4 in regulating both amelogenin expression and exon4 splicing, leading to enamel defects when absent.

**Supplementary Information:**

The online version contains supplementary material available at 10.1038/s41598-026-40706-0.

## Introduction

The amelogenin gene is highly conserved across species and encodes proteins essential for enamel formation, serving as the primary structural matrix during tooth development^1^. In humans, amelogenin is present on both the X and Y chromosomes (*AMELX*/*AMELY*), with over 90% of transcripts derived from *AMELX*^1,2^. Mutations in *AMELX* cause X-linked Amelogenesis Imperfecta (AI), a condition characterized by defective enamel formation. In mice and rats, amelogenin is located solely on the X chromosome (*Amelx*). In both humans and mice, amelogenin mRNA undergoes extensive alternative splicing, generating up to 17 transcript variants^1,3^. Among these events, exon4 splicing is pivotal to form two predominant isoforms: a long-form (M180 in mice, H174 in humans) critical for enamel matrix formation, and a short-form (LRAP) involved in signaling^3–6^. Both lack exon4, though this exon is highly conserved across species^7^.

Despite being mostly excluded from protein-coding transcripts, the spliced-out exon4 gives rise to a microRNA, miR-exon4^8^. Unlike the well-characterized protein isoforms, the function of miR-exon4 remains to be discovered. Our previous studies demonstrated that miR-exon4 regulates *Runx2*, a key transcription factor in enamel and bone development^9–12^, by directly targeting *Nfia* and *Prkch* in ameloblasts and osteoblasts^13^. Furthermore, mutations in *Amelx* exon4 or exon5 causing X-linked AI correlate with reduced exon4 splicing and diminished miR-exon4 production *in vitro*^14^. These findings highlight the dual significance of exon4 splicing in producing the proper amelogenin protein isoforms and ensuring miR-exon4 generation, with implications for both normal and pathologic enamel development. A loss of miR-exon4 may therefore represent one of the molecular etiologies underlying X-linked AI.

This study examines the *in vivo* role of miR-exon4 in enamel formation and investigates its potential involvement in exon4 splicing, offering new insights into its role in the development of X-linked AI.

## Results

### miR-exon4-*Nfia*/*Prkch*-*Runx2* axis is active *in vivo*

**Fig. 1:**
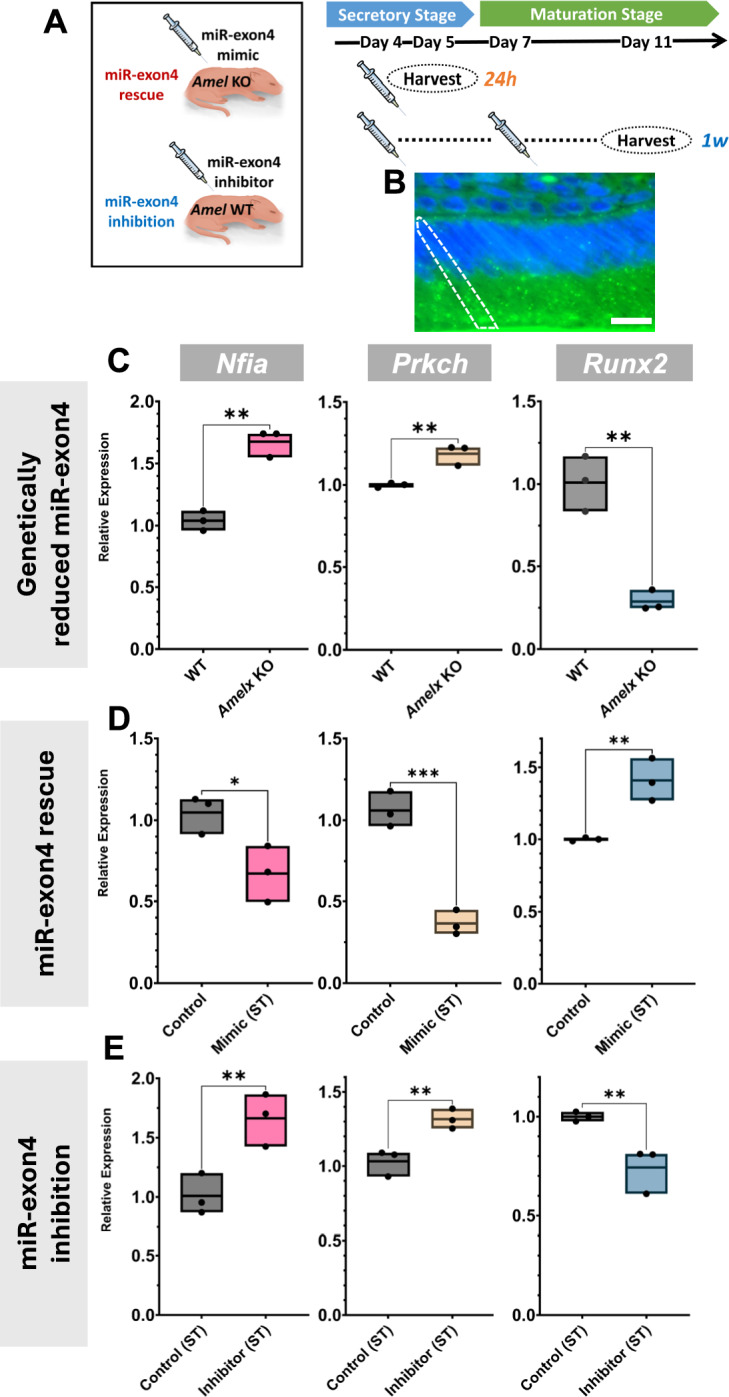
Changes in available miR-exon4 affect the direct and indirect targets of miR-exon4 in the enamel organ. **A**) Experimental design for the 24-hour and 1-week models of miR-exon4 inhibitor and mimic injection. **B**) FITC labels in green signals detected in the ameloblast layer confirm oligonucleotide delivery to the ameloblasts. One ameloblast is outlined with a dashed line. Nuclei are seen in the blue signal. Bar: 25 µm. **C**-**E**) qPCR analysis for *Nifa*, *Prkch,* and *Runx2.* In the postnatal day 5 (P5) *Amelx* KO enamel organ, where miR-exon4 production is genetically reduced, mRNA expression of known miR-exon4 direct targets (*Nfia* and *Prkch*) increased, and a further downstream target (*Runx2*) decreased (**C**). 24 hours after miR-exon4 mimic rescue to the *Amelx* KO, mRNA expression of *Nfia* and *Prkch* decreased, and *Runx2* increased in the enamel organ (**D**). Conversely, 24 hours after miR-exon4 inhibition in the *Amelx* WT mice resulted in the upregulation of *Nfia* and *Prkch* and the downregulation of *Runx2,* similar to the *Amelx* KO mice (**E**). Each box represents the minimum to maximum data collected (shown as individual dots), with the middle line indicating the mean. n=3 per group. *: p<0.05 and **: p<0.01.

In previous *in vitro* studies, we found that miR-exon4 functions by directly targeting *Nfia* and *Prkch*, leading to the downregulation of their mRNA expression, which increases downstream *Runx2* expression. To confirm whether this regulatory axis is active *in vivo*, we examined the enamel organ of the first molars from three animal models: 1) a model with genetically reduced miR-exon4 production (*Amelx* knockout/KO mice), 2) a miR-exon4 rescued model (*Amelx* KO mice supplemented with miR-exon4 mimic), and 3) a miR-exon4 loss-of-function model (*Amelx* wild-type/WT mice treated with a miR-exon4 inhibitor), with mimic and inhibitor injections administered 24 hours prior to analysis at postnatal day 5 (Fig. 1A). Delivery of LNA (Locked Nucleic Acid) oligonucleotides to ameloblasts was verified using a FITC-tagged universal negative control oligonucleotide (Fig. 1B). In *Amelx* KO mice, *Nfia* and *Prkch* levels were significantly upregulated, while *Runx2* was downregulated compared to WT (Fig. 1C). Supplementation with miR-exon4 in KO mice resulted in the significant downregulation of *Nfia* and *Prkch* and the upregulation of *Runx2* compared to the control (Fig. 1D). Conversely, inhibition of miR-exon4 in *Amelx* WT mice significantly upregulated *Nfia* and *Prkch*, with a corresponding downregulation of *Runx2* (Fig. 1E). These data support the existence of a regulatory axis involving miR-exon4, *Nfia*/*Prkch*, and *Runx2* in the *in vivo* enamel organ, consistent with our prior *in vitro* findings.

Although it is difficult to selectively measure endogenous mature miR-exon4 levels with exogenous mimic and inhibitor injections, neither treatment significantly changed precursor miR-exon4 levels, indicating that endogenous miR-exon4 production was unaffected by the exogenous miR-exon4 mimic and inhibitor (Appendix Fig. 1).

In long-term (1 week) inhibition of miR-exon4 starting at postnatal day 4, the effects on *Prkch* and *Runx2* were consistent with short-term (24 hours) inhibition. However, *Nfia* expression became unresponsive to miR-exon4 inhibitor (Appendix Fig. 2). This difference in *Nfia* response suggests a developmental stage-specific interaction with miR-exon4, similar to what we previously observed in osteoblast cultures^13^.

### miR-exon4 inhibition resulted in an enamel mineralization defect

**Fig. 2:**
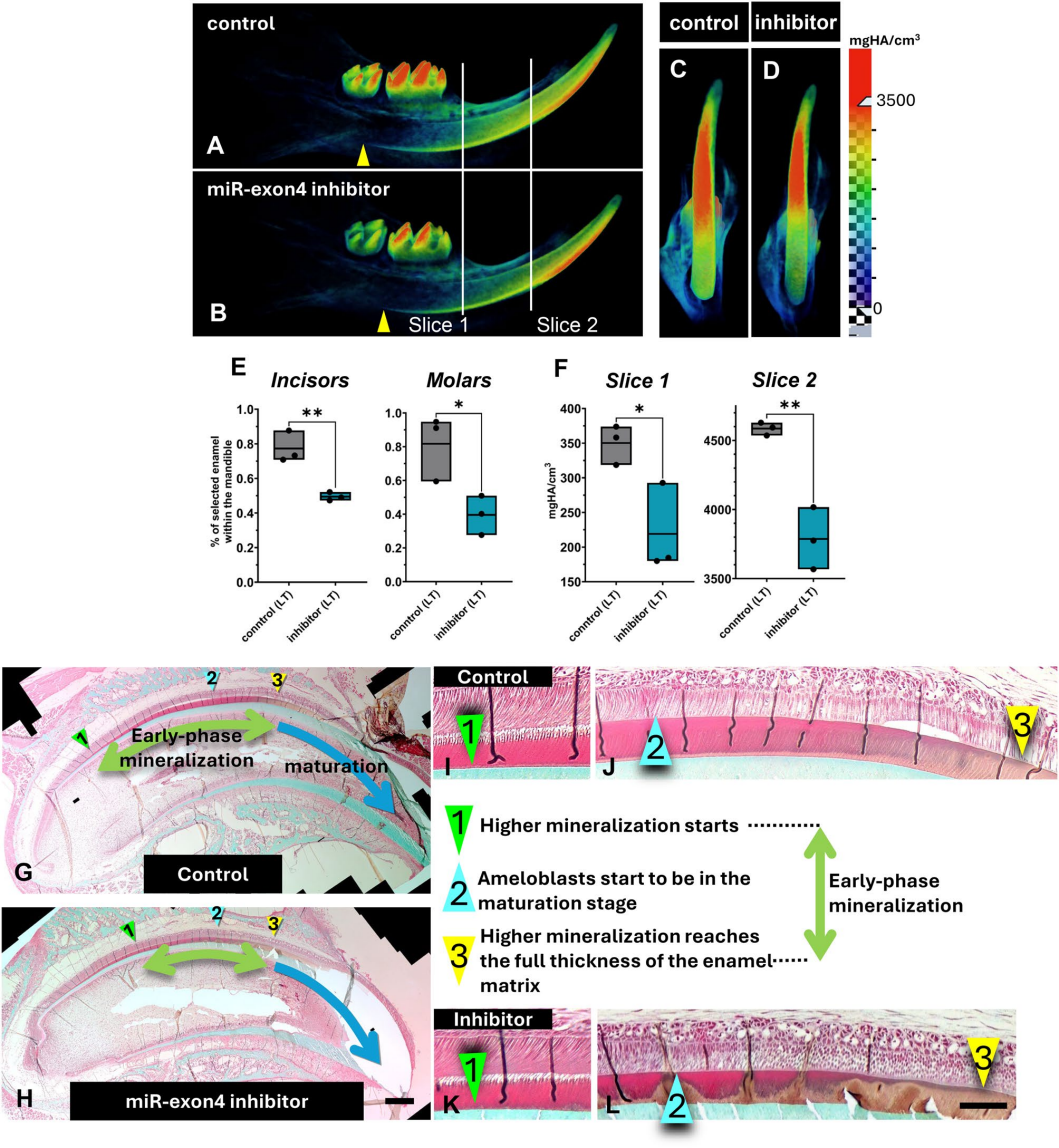
Effect of long-term (1-week) miR-exon4 inhibition on enamel mineralization. **A**–**F**) Micro-CT analysis on mandibles. Heat maps of mineral density acquired shows a reduction in the area of highly mineralized tissue (indicated in red) in the inhibitor-injected group (**A**–**D**). The delay at the start of mineralization (arrowheads) is also observed in this group (**A** and **B**). The volume percentage of areas with a mineral density greater than 3500 HA mg/cm^3^ within the mandible reveals a significant decrease in mineralization in the incisors and molars of the inhibitor-injected group (**E**). Quantification of enamel mineral density at two slice locations (**A** and **B**) indicates decreased mineral density in the inhibitor-injected group (**F**). Each box plot displays the range of data (individual dots), with the middle line representing the mean. n=3 per group *: p<0.05, **: p<0.01 **G** and **H**) Trichrome-stained undecalcified maxillary incisor sections from control and miR-exon4 inhibitor-treated mice show enamel mineralization progression. In controls, mineralization initiates at the dentin-enamel junction (DEJ) and spreads toward the enamel surface through maturation stages. Bar: 1 mm **I–L**) Close-up views of panels G and H, where the beginning and end of early mineralization are marked by brownish staining (arrowheads 1, **I** and **K**) until it spans the entire enamel thickness (arrowheads 3, **J** and **L**). The onset of ameloblast maturation is defined based on cellular morphology (arrowheads 2). Bar: 100 µm. Altogether, inhibitor-treated samples show delayed and shorter early-phase mineralization (summarized on **G** and **H**), indicating impaired enamel development.

As RUNX2 is critical for enamel mineralization, to assess the effect of miR-exon4 on enamel mineralization, mandibular incisors and molars from the long-term treatment group were analyzed using micro-CT scanning. The mineral density heat map showed a clear decrease in mineralization in the inhibitor-injected mice compared to controls (Fig. 2A-D). The proportion of mature enamel volume with mineral density above 3500 mg HA/cm^3^ was significantly lower in mice treated with the miR-exon4 inhibitor, in both incisors and molars (Fig. 2E). Heat maps also indicated a noticeable delay in the start of mineralization in incisor enamel (Fig. 2A and B). Since these teeth had not yet erupted, the mineralization defects observed are likely due to the biological effects of miR-exon4 inhibition. The impact of the miR-exon4 inhibitor on enamel mineralization was further confirmed at earlier stages, as shown by reduced enamel density in incisor slices at the locations indicated in Fig. 2A and B (Fig. 2F).

3D tomography (Appendix Fig. 3) revealed that the inhibitor-injected mice had rough enamel surfaces on both incisors and molars compared to controls, and the incisor enamel had a less distinct boundary with the root analog surface cementum. Overall, this suggests defective enamel mineralization caused by miR-exon4 inhibition.

To evaluate enamel mineralization progress, Trichrome staining was performed on undecalcified maxillary incisor sections. Enamel with higher mineralization appeared grayish-brown, distinguishable from the red-pink staining of less mineralized, organic-rich enamel matrix^15^. The border between these two conditions of the enamel matrix is recognized as the front line of mineralization. By tracking this border, we defined early-phase mineralization as the border that starts at the enamel-dentin junction (Fig. 2I and K, arrowheads 1) and extends until full thickness is achieved (Fig. 2J and L, arrowheads 3). We found that treatment with the miR-exon4 inhibitor delayed the onset of early-phase mineralization (Fig. 2G and H), which is consistent with micro-CT results. Notably, the point marking the start of the maturation stage (Fig. 2G, H, J, and L, arrowheads 2) remained unchanged in the treated mice, indicating that miR-exon4 inhibition shortened the duration of the early-phase mineralization.

### miR-exon4 regulates RUNX2 to further regulate amelogenin expression

RUNX2 is a key transcription factor in enamel formation, as evidenced by enamel defects in *Runx2*-deficient mice and in humans with *RUNX2* mutations^10–12^. In ameloblasts, RUNX2 negatively regulates *Amelx* expression^16^. Given that miR-exon4 inhibition reduced both *Runx2* expression and enamel mineralization, we next examined RUNX2 and amelogenin protein levels in ameloblasts of maxillary incisors from long-term treated mice.

**Fig. 3:**
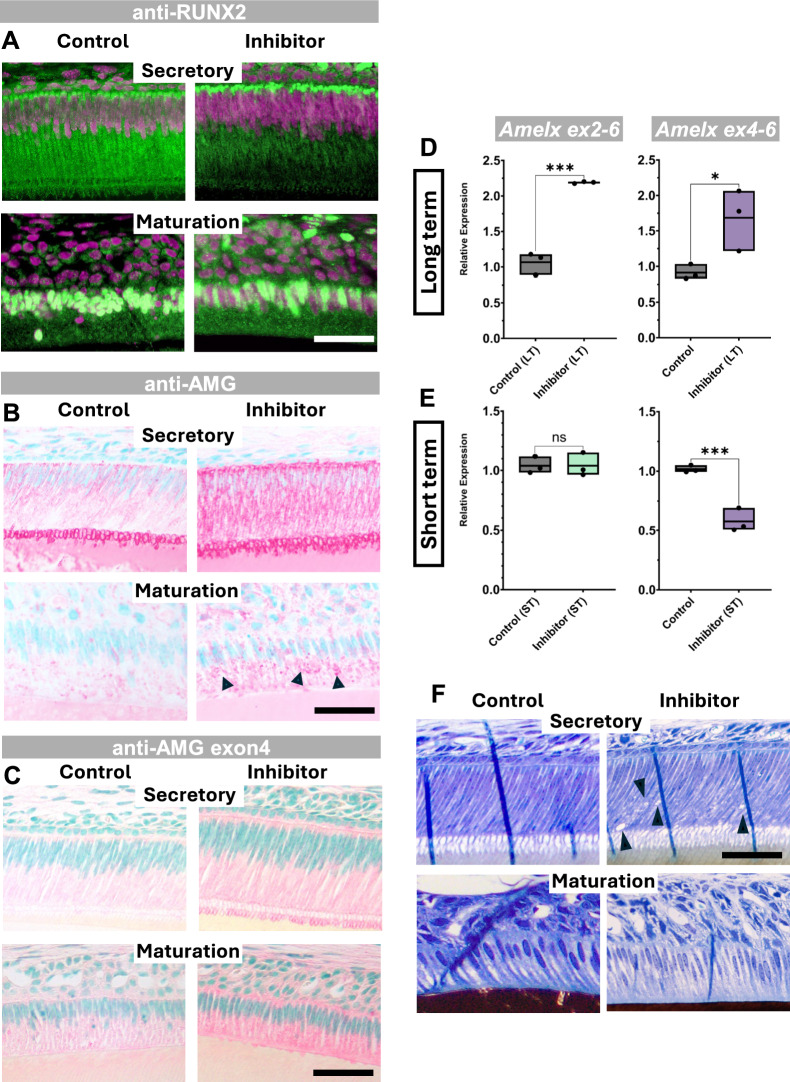
Effect of miR-exon4 inhibition on RUNX2, amelogenin protein, and mRNA expression. **A**-**C**) Immunohistochemistry on maxillary incisors of long-term (1-week) treated mice. RUNX2 immunostaining, seen in green fluorescent dots, is observed in both the cytoplasm and nucleus of ameloblasts (**A**). The staining predominantly appears in the cytoplasm during the secretory stage and in the nucleus during the maturation stage. Long-term inhibition of miR-exon4 appears to reduce RUNX2 immunostaining in both secretory and maturation ameloblasts (**A**). Inhibition of miR-exon4 significantly increases immunostaining of amelogenin proteins (AMG) in the cytoplasm of ameloblasts at both the secretory and maturation stages, detected with anti-long-form AMG antibodies (**B**). A noticeably stronger signal appears in the Golgi apparatus region of maturation ameloblasts in the inhibitor group (arrowheads) (**B**). Immunostaining with anti-AMG exon4 antibody also shows a clear increase in signal in ameloblasts at both stages in the inhibitor-treated group (**C**). **D**-**E**) qPCR analysis of total *Amelx* and isoforms containing exon4. Long-term inhibition of miR-exon4 shows upregulation of both the mRNA levels of entire amelogenin transcripts, detected by the exon2-exon6d primer set, and *Amelx* isoforms containing exon4 (**D**). Short-term (24-hour) miR-exon4 inhibition does not alter total *Amelx* expression but specifically affects *Amelx* isoforms with exon4 (**E**). Each box depicts the range of data (represented as individual dots), with the middle line indicating the mean. n=3 per group; * p<0.05, *** p<0.001. F) Toluidine blue and Von Kossa staining of undecalcified plastic sections show no major disruption in ameloblast morphology or arrangement in the inhibitor-injected group at both stages. However, vacuole structures (arrowheads) develop in secretory ameloblasts after the injection of an inhibitor. Bars: 50 µm.

In control mice, RUNX2 was detected in both the cytoplasm and nucleus of secretory ameloblasts, with predominant cytoplasmic localization. By the maturation stage, the majority of RUNX2 is localized to the nucleus. MiR-exon4 inhibition decreased RUNX2 staining at both stages, consistent with mRNA downregulation (Fig. 3A). While RUNX2 protein is well documented in maturation ameloblasts, its presence and cytoplasmic retention in the secretory stage have not been widely reported. To further validate this observation, we examined RUNX2 subcellular localization in LS8 ameloblast-like cells (which model secretory-stage ameloblasts^17^) and reviewed the mRNA expression atlas based on nine published scRNA-seq datasets of mouse ameloblasts^18^. We confirmed cytoplasmic and nuclear RUNX2 localization in LS8 cells (Appendix Fig. 4A), consistent with its known cytoplasmic sequestration in osteoblasts when bound to STAT1 or prevented from nuclear translocation^19,20^. *Runx2* mRNA was also clearly expressed in secretory ameloblast clusters (Appendix Fig. 4B). These data suggest that RUNX2 is present in secretory ameloblasts as transcriptionally less active state, supporting higher *Amelx* expression during this stage than the maturation stage^8^.

Given that RUNX2 suppresses *Amelx*, its downregulation by miR-exon4 inhibition is expected to enhance amelogenin protein (AMG) expression. Indeed, we observed increased cytoplasmic AMG staining in secretory ameloblasts and in the Golgi region of maturation-stage cells (Fig. 3B), indicating elevated synthesis. Isoforms containing exon4 also increased in both stages, as shown by anti-AMG exon4 antibody staining (Fig. 3C). At the mRNA level, both total *Amelx* transcripts (exon2–6d primers) and exon4-containing isoforms (exon4–6d primers) were upregulated (Fig. 3D), mirroring the overexpression of long-form amelogenin (M194) known to impair enamel formation in TgM194 mice^21^.

Interestingly, the short-term (24-hour) miR-exon4 inhibition decreased the mRNA isoforms with exon4 without affecting the total *Amelx* expression (Fig. 3E), indicating increased exon4 splicing. Even in the long-term treatment group, miR-exon4 inhibition promoted exon4 splicing, as shown by the smaller increase in exon4-containing isoforms relative to total *Amelx* (Fig. 3D). These findings suggest that miR-exon4 simultaneously regulates *Amelx* transcription through modulation of RUNX2, as well as exon4 splicing.

Toluidine Blue staining, followed by Von Kossa staining of undecalcified sections, showed no significant change in overall ameloblast morphology or arrangement due to miR-exon4 inhibition. However, secretory stage ameloblasts in inhibitor-treated mice exhibited vacuolar structures similar to those observed in compromised ameloblast function that leads to enamel defects^22^ (Fig. 3F). This clearly demonstrates the disruption of secretory ameloblast function following miR-exon4 inhibition.

### miR-exon4 regulates mRNA expression of SRSFs that are possibly associated with exon4 splicing

**Fig. 4:**
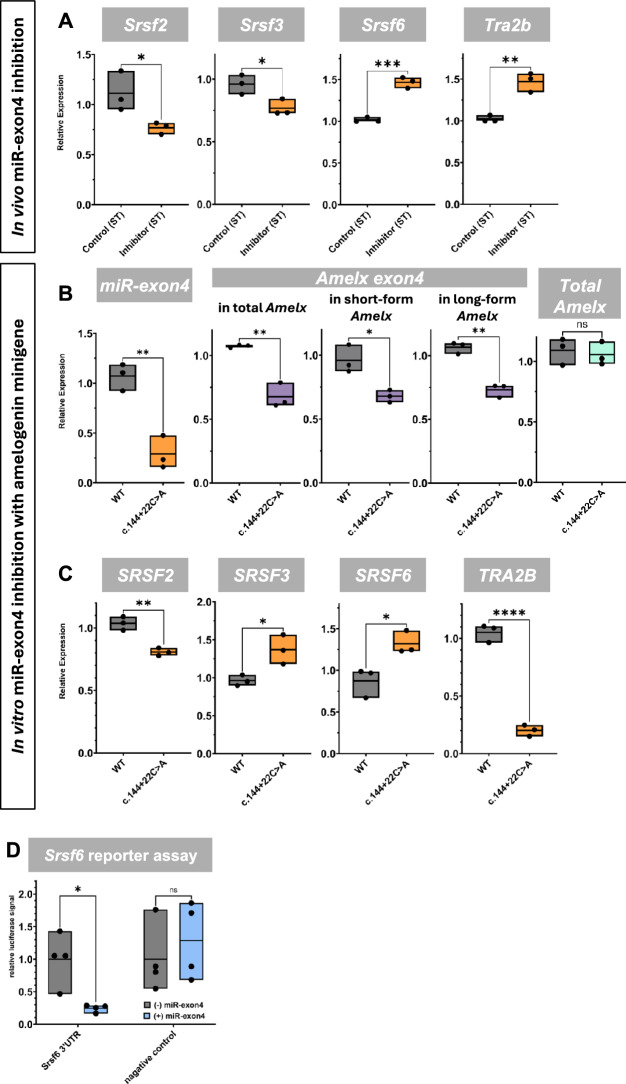
Effect of miR-exon4 inhibition on splicing regulators. **A**-**C**) qPCR analysis on mRNAs for *Srsf2, Srsf3, Srsf6*, and *Tra2b. In vivo,* short-term (24-hour) miR-exon4 inhibition of the enamel organ shows a significant change in the mRNA expression of *Srsf2, Srsf3, Srsf6*, and *Tra2b* (**A**). *In vitro* with HEK293 cells, reduced miR-exon4 production from the *Amelx* minigene by the c.144+22C>A mutation also leads to a significant reduction in miR-exon4 production (**B**). Under these conditions, the inclusion of exon4 within major *Amelx* mRNA isoforms is significantly decreased, while overall *Amelx* production remains unchanged (**B**). *In vitro*, miR-exon4 inhibition similarly results in significant changes in mRNA expression of *SRSF2, SRSF3, SRSF6*, and *TRA2B* (**C**). **D**) Luciferase reporter assay with the *Srsf6* 3’UTR reporter. miR-exon4 mimic significantly suppresses luciferase activity, whereas the negative control vector shows no response to miR-exon4. Each box indicates the range from minimum to maximum data points (shown as individual dots), with the middle line representing the mean. n=3 per group. *: p<0.05, **: p<0.01, ***: p<0.001.

Since the loss of functional miR-exon4 altered the alternative splicing of amelogenin exon4, it suggests that miR-exon4 may play a role in exon4 splicing. Ser/Arg-rich splicing factors (SRSFs) are crucial regulators of alternative splicing, determining whether exons are included or excluded. To explore how miR-exon4 regulates *Amelx* exon4 alternative splicing, we updated our computational prediction of miR-exon4 targets from our previous study^13^ using the newer version of the DIANA tool (https://dianalab.e-ce.uth.gr/html/universe/index.php?r=mrmicrot/index)^23^. This analysis identified 10 SRSFs (SRSFs 2, 3, 6, 7, 8, 9, 10, 11, 12, and TRA2B) as potential direct targets of miR-exon4 (Appendix Fig. 5). Among them, we previously reported that SRSF2 binds to exon4^14^. Further in silico prediction using ESE-Finder Ver 3.0 (https://esefinder.ahc.umn.edu/cgi-bin/tools/ESE3/esefinder.cgi)^24^ and RBP map (https://rbpmap.technion.ac.il/)^25^ suggested that SRSFs 3 and 6 potentially bind to exons 4 and 5, while TRA2B binds to exon5, all potentially regulating exon4 splicing. Therefore, we measured mRNA levels of *SRSF*s 2, 3, 6, and *TRA2B* in mouse samples treated with a short-term inhibitor. This analysis revealed significant upregulation of *Srsf6* and *Tra2b* and downregulation of *Srsf2* and *Srsf3* (Fig. 4A). To further confirm the effects of reduced miR-exon4 on *Amelx* exon4 splicing and *SRSF* expression, we introduced a genetic mutation (c.144+22C>A) in the *Amelx* minigene to reduce miR-exon4 biosynthesis. This mutation disrupts the CNNC sequence in intron4, which is crucial for miR-exon4 maturation as the Drosha recruiting site, thereby blocking mature miR-exon4 biosynthesis from the spliced-out exon4 without affecting the mRNA coding sequence^8,26^. Transfection of WT and c.144+22C>A minigenes into HEK293 cells confirmed reduced miR-exon4 biosynthesis (Fig. 4B). Similar to *in vivo* miR-exon4 inhibition, the c.144+22C>A mutation decreased exon4 inclusion within total amelogenin mRNA while maintaining overall *Amelx* transcription driven by the CMV promoter, indicating increased exon4 splicing (Fig. 4B). Previously, we reported a possible correlation between exon4 splicing and the formation of long- and short-form amelogenin via exon6abc splicing^14^. With this c.144+22C>A mutation, we observed increased exon4 splicing occurring equally in both the short-form and long-form amelogenin mRNAs, confirming the effectiveness of reduced miR-exon4 on exon4 splicing *in vitro* (Fig. 4B). Examining *SRSF* mRNA levels, we found that *SRSFs 3* and *6* were significantly upregulated, while *SRSF2* and *TRA2B* were downregulated (Fig. 4C). Since miRNAs typically act as negative regulators of their direct targets, the consistent upregulation of *SRSF6* upon miR-exon4 inhibition, both *in vitro* and *in vivo*, suggests a potential direct regulation by miR-exon4. This was further supported by reporter assays using *Srsf6* 3’UTR and miR-exon4 (Fig. 4D). The significant changes observed in other *SRSF*s also suggest indirect regulation by miR-exon4. These findings imply that miR-exon4 may influence Amelx exon4 splicing, possibly by altering *SRSF* gene expression. While our data shows consistent RNA-level changes, we interpret these findings as correlative rather than definitive proof of a mechanistic role.

### miR-exon4 is present in the nucleus to bind amelogenin pre-mRNA

Since alternative splicing is a fundamental cellular process, any changes in SRSF expression typically have widespread effects on splicing across many genes, potentially causing systemic impacts. However, the relatively specific phenotypes observed in our miR-exon4 inhibition models suggest that miR-exon4 may selectively influence the splicing of *Amelx* exon4. This indicates that miR-exon4 could directly and specifically contribute to the alternative splicing of exon4.

**Fig. 5:**
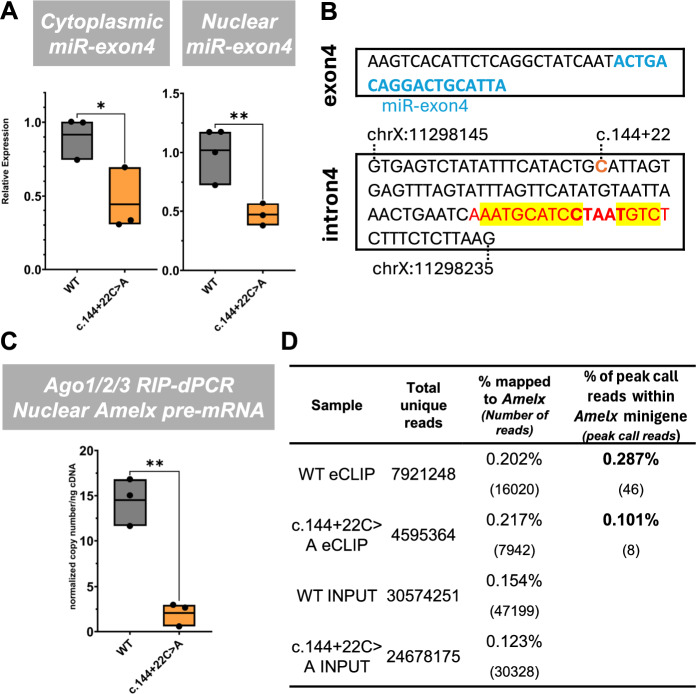
Nuclear miR-exon4 binding to *Amelx* pre-mRNA. **A**) qPCR analysis of miR-exon4 in HEK293 cells after WT and c.144+22C>A mutated *Amelx* minigene transfection. A significant reduction in miR-exon4 production caused by the c.144+22C>A mutation is clearly visible in both cytoplasmic and nuclear RNA fractions. **B**) RNA hybrid prediction between *Amelx* intron4 and miR-exon4. The predicted binding site in the intron4 sequence is shown in red. The yellow highlight indicates the actual hybrid sequence. The red bold sequence indicates the branch point sequence (YTNAY). **C**) Ago1/2/3 RIP on the nuclear RNA of HEK293 cells after WT and c.144+22C>A mutated *Amelx* minigene transfection. Measurement of *Amelx* pre-mRNA by digital PCR with intron5-exon6d primers shows that the decrease in miR-exon4 with the c.144+22C>A mutation also resulted in a significant reduction in nuclear *Amelx* pre-mRNA. Each box represents the range from minimum to maximum data points (shown as individual dots), with the middle line indicating the mean. n=3 per group. *: p<0.05, **: p<0.01, ***: p<0.001. D) eCLIP-seq of HEK293 cells transfected with WT and c.144+22C>A mutation *Amelx* minigene. Region-specific peak call analysis identified a peak at the predicted 18-nt miR-exon4 binding site+2nt in *Amelx* intron4 (chrX:167,965,539–167,965,556) in the WT sample. This peak was markedly decreased in the c.144+22C>A mutant. Comparing the WT and c.144+22C>A mutant, read depths in input samples and *Amelx* reads in eCLIP samples are similar, indicating that the reduced peak reads in the mutant result from the loss of miR-exon4 caused by the mutation.

While miRNAs traditionally act in the cytoplasm to suppress target mRNAs’ stability or protein production, they are also known to function in the nucleus. There, they bind to RNA and DNA through the miRNA-Ago complex to regulate transcription, RNA stability, and even alternative splicing^27,28^. To explore whether miR-exon4 functions in the nucleus, we analyzed subcellular RNA fractions in the same cell culture model using the *Amelx* minigene. Transfecting HEK293 cells with WT *Amelx* minigenes confirmed the presence of miR-exon4 in the nuclear RNA fraction (Fig. 5A). The c.144+22C>A mutation significantly decreased miR-exon4 levels in both the cytoplasmic and nuclear RNA fractions (Fig. 5A). If miR-exon4 directly influences the alternative splicing of *Amelx* exon4, its potential targets are likely found between exons 3 and 5 of amelogenin pre-mRNA, the region crucial for defining exon4 splicing^14^. Using computational RNA hybrid prediction (https://bibiserv.cebitec.uni-bielefeld.de/rnahybrid/)^29^, we found that miR-exon4 can potentially hybridize specifically with intron4 at the branch point sequence, a crucial site for exon4 splicing, shown by Splice-Alternative Profile Predictor (SpliceAPP, https://bc.imb.sinica.edu.tw/SpliceAPP/) utilizing the SVM BP Finder algorithm^30^ (Appendix Fig. 6 and Fig. 5B). This branch point is recognized by splicing factor 1 (SF1), which recruits the U2 spliceosome to initiate splicing at the 3’ end of the splice site. If miR-exon4 binds to this branch point, it could potentially compete with SF1, inhibiting exon4 splicing. Conversely, inhibiting miR-exon4 could free this branch point for SF1, thereby promoting exon4 splicing.

To test whether miR-exon4 binds to *Amelx* pre-mRNA in the nucleus, we used RNA immunoprecipitation (RIP) with an anti-Ago1/2/3 antibody to isolate nuclear RNA bound to the Ago-miRNA complex from HEK293 cells transfected with either the *Amelx* WT or the c.144+22C>A minigene. We then detected the presence of *Amelx* pre-mRNA in the precipitated samples. The amount of pulled-down *Amelx* pre-mRNA was significantly lower in cells with the c.144+22C>A mutant minigene compared to WT (Fig. 5C), corresponding to the reduced level of nuclear miR-exon4 in the c.144+22C>A mutant (Fig. 5A). This suggests that miR-exon4 directly targets *Amelx* pre-mRNA within the nucleus.

To further identify the location of miR-exon4 binding in *Amelx* pre-mRNA, we performed enhanced crosslinking and immunoprecipitation (eCLIP) on HEK293 cells transfected with either the *Amelx* WT or the c.144+22C>A minigene, using the same anti-Ago1/2/3 antibody. Although several peak-calling algorithms were tested, including PureCLIP (v1.3.2), no confident peak could be assigned in intron4 due to confounding factors—such as a strong neighboring crosslinking site in exon5 and a predominance of exonic reads from whole-cell lysates—both of which suppressed sensitivity in intronic regions. To address this, we employed a region-specific filtering approach that directly quantifies reads overlapping the predicted 18-nt miR-exon4 binding site at the intron4 branch point. This customized approach enabled the identification of Ago binding that standard peak calling overlooks. In the total eCLIP mapped reads, the proportion of *Amelx* mapped reads was similar between WT and c.144+22C>A minigene sample. In WT eCLIP samples, peak calling showed Ago1/2/3 binding at the predicted intron4 branch point region (Appendix Fig.7A). In contrast, the c.144+22C>A sample showed a marked decrease in peak reads at this site, with the % of *Amelx* minigene reads dropping from 0.250% to 0.101% (Fig. 5D). These findings support a direct interaction between miR-exon4 and the intron4 branch point sequence of *Amelx* pre-mRNA.

## Discussion

This study shows that miR-exon4, a miRNA derived from the splicing of *Amelx* exon4, plays an essential regulatory role in enamel formation through both direct and indirect mechanisms. Our results suggest that miR-exon4 functions via the miR-exon4–*Nfia*/*Prkch*–*Runx2* pathway and further influences *Amelx* expression while also regulating the alternative splicing of *Amelx* exon4 *in vivo*.

### Significance of Amelx exon4 alternative splicing

Exon4 splicing in *Amelx* is crucial for producing two major mRNA isoforms and generating miR-exon4 from the spliced-out exon4^8^. Although typically excluded, exon4 is occasionally retained to produce protein-coding transcripts. In mice, exon4-containing *Amelx* mRNAs are expressed in ameloblasts throughout differentiation, with varying levels^8^. The resulting protein (AMG+ex4) is mainly seen during the maturation stage, indicating a stage-specific role^15^. Additionally, functional differences between AMG+ex4 and -ex4 have been demonstrated both *in vivo* and *in vitro*^4,31,32^. In fact, the overexpression of the AMG+ex4 isoform (M194) at all stages results in enamel defects similar to those seen in human X-linked conditions AI^21^, indicating that exon4 inclusion requires stage-specific regulation. Prior studies further implicate miR-exon4 in enamel development^8,13^, particularly in the possibility of miR-exon4 reduction in developing enamel defects within X-linked AI associated with altered exon4 splicing^14^. These highlight the importance of exon4 alternative splicing in diversifying proper amelogenin functions and its disruption resulting in some forms of X-linked AI.

### Potential dual role of miR-exon4 in regulating amelx splicing

We previously reported the potential role of SRSF2 and SRSF5 in the alternative splicing of exon4. Here, we identified mRNAs for SRSF2, SRSF3, SRSF6, and TRA2B as regulated by miR-exon4, both directly and indirectly; these are predicted to bind exons 4 and 5. Although SRSF binding is the key to determining whether alternative exons are included or skipped, the effects of changes in SRSF expression on exon-specific binding are not fully understood. Additional research is needed to clarify how each SRSF interacts with specific *Amelx* exons. Our RNA-level data using *Amelx* minigene to quantitatively detect exon4 splicing, as well as *in vivo* model, provide strong correlative evidence; definitive mechanistic conclusions associated with SRSF contribution will require protein-level assays and further eCLIP-seq in future studies. Nonetheless, our findings on the direct regulation of *Srsf6* by miR-exon4 support the notion that miR-exon4 is potentially involved in *Amelx* exon4 alternative splicing via SRSFs.

The role of nuclear miRNAs is a developing research area, and our confirmation of miR-exon4’s presence in the nucleus marks an important step in understanding its nuclear function. Our eCLIP and RIP-dPCR results show that nuclear miR-exon4 associates with Ago2 complexes bound to *Amelx* intron4 at the branch point. This direct interaction is disrupted in the c.144+22C>A mutant with lower miR-exon4 production, further supporting a nuclear role for miR-exon4 in regulating *Amelx* pre-mRNA.

MicroRNAs are known to interact with transcription factors and regulate gene expression in a stage-specific manner, often forming feedback loops during development^33,34^. This mechanism is observed across species, from plants to mammals, including in the craniofacial development of mice^35,36^. The regulatory role of miR-exon4 in exon4 alternative splicing aligns with these well-established principles of miRNA function. Given the importance of *Amelx* exon4 splicing for producing key derivatives essential for enamel formation, further research is necessary to understand how miR-exon4 interacts with the splicing machinery to regulate exon4 splicing, thereby improving our knowledge of amelogenesis.

### miR-exon4 influences enamel mineralization and amelx expression

In this study, inhibiting miR-exon4 *in vivo* shortened the early-phase mineralization period and decreased enamel mineral density, supporting a functional link between miR-exon4 reduction and the development of amelogenesis imperfecta. The research also found that miR-exon4 inhibition increased the expression of both amelogenin mRNA and protein, including isoforms that contain exon4 (AMG+exon4). Although the precise mechanism by which the upregulated amelogenin protein isoforms alter enamel mineralization remains unclear, existing findings offer valuable insights. In mice, overexpression of amelogenin lacking exon4 (M180) does not cause enamel defects, whereas overexpression of the isoform with exon4 (M194) does^21,37,38^. Excessive AMG+exon4 could thus directly contribute to impaired enamel mineralization when miR-exon4 is inhibited. Since amelogenin isoforms are produced in a stage-dependent manner^8^, these findings indicate that miR-exon4 is essential for maintaining the proper balance of amelogenin isoforms during enamel development to ensure correct enamel mineralization.

### miR-exon4 downstream axis and broader target network

Our findings confirm that miR-exon4-*Prkch*/*Nfia*-*Runx2* functions in the enamel organ *in vivo*, with *Nfia* responding to miR-exon4 inhibition in a stage-specific manner. These results indicate that miR-exon4 exerts temporal control over key regulators of ameloblast function. Supporting this, our previous proteomics analysis using Ingenuity® Pathway Analysis in MC3T3-E1 cells lacking miR-exon4 (M14-mi2), which showed 68 upregulated molecules (fold change >1.5) with 36 identified as potential direct targets of miR-exon4— including *Prkch*^13^—revealed that 9 of these 36 candidates are associated with 16 hereditary enamel defect (HED) molecules reported by Wright et al., linked to genetic loci for 71 HED conditions^39^. This pathway analysis suggests that miR-exon4 may play a role in enamel formation by regulating a broader network.

## Conclusion

While these results do not yet establish the full landscape of miR-exon4-mediated regulation, they support a model in which miR-exon4 modulates *Amelx* splicing and expression both through splicing factor regulation and direct RNA interactions, thereby contributing to enamel mineralization. Ongoing studies will be needed to determine whether additional targets or pathways contribute to the enamel phenotype associated with miR-exon4 inhibition.

## Materials and methods

### Animal

*Amelx* WT and KO mice^40^ colonies were gifted by Dr. Carolyn Gibson, University of Pennsylvania, and were maintained at the UCSF LARC (Laboratory Animal Research Center). All animal experiments were approved by the University of California, San Francisco (UCSF) Institutional Animal Care and Use Committee (IACUC; Protocol No. AN200166-00). All methods were performed in accordance with relevant institutional and national guidelines and regulations, including the ARRIVE 2.0 guidelines.

Pups from the same litter were randomly split into 4 groups: experimental short-term, experimental long-term, vehicle control short-term, and vehicle control long-term. A litter’s size defined each group’s sample size (n=3). The sex of the pups was randomly allocated within the group. At postnatal day 4 (P4) and P7, the pups received mirVana miR-exon4 mimic oligonucleotide (1 nmol/gBW, Thermo Fisher Scientific, Waltham, MA), miRCURY LNA™ miR-exon4 inhibitor oligonucleotide (10 nmol/gBW, Qiagen, Hilden, Germany), or vehicle control by intraperitoneal injection. The average body weight of the animals beginning the experiment at P4 was *Amelx* WT 3.10 g (SD=0.42) and *Amelx* KO 2.16 g (SD=0.29). The FITC-labeled universal negative miRNA inhibitor control oligonucleotide was injected to confirm oligonucleotide delivery to the ameloblasts. The dosage was determined by the manufacturer’s instructions. At P5 (after 24 hours of primary injection) or at P11 (after 1 week of primary injection), mice were euthanized by CO_2_ inhalation followed by decapitation, and mandibles and maxillae were dissected for the subsequent sample harvest (Fig. 1A).

### RNA extraction and qPCR

Enamel organs of the first molars were extracted from the dissected-out mandibles and maxillae and processed for the total RNA extraction using the Direct-zol RNA miniprep plus kit (Zymo Research, Irvine, CA). cDNA reverse transcribed from mRNA was obtained using Superscript IV VILO First-Strand Synthesis Master Mix for qPCR (Thermo Fisher).

Expression of mRNAs was examined by qPCR with TB Green Premix Ex Taq II (Takara Bio, Shiga, Japan) using QuantStudio™ 6 Pro (Thermo Fisher) with primer sets for *Runx2, Prkch, Nfia, Amelx* exon4*-6d, Srsf’s 2, 3, 6, and Tra2b* (Elim Biopharmaceuticals, Hayward, CA). *Amelx* exon2-PA28956^41^ and exons 2-6d were amplified using the KOD SYBR qPCR mix (Toyobo, Osaka, Japan). *Mrpl19* was used as a reference gene. Primer sequences are listed in Appendix Table 1. The Cq value of each qPCR amplification was obtained by Design and Analysis software (Thermo Fisher). The relative expression levels of target genes were analyzed using the ΔΔCt method^42^. The expression of each gene was calculated as a relative expression level (fold change) compared with vehicle control or WT vs *Amelx* KO. The significance of differences was determined by the independent Student’s t-tests following the F-test^43^ using GraphPad Prism software (ver. 10.4.2, GraphPad Software, Boston, MA, https://www.graphpad.com/). p<0.05 was considered significantly different.

### Micro-CT scanning and analysis

The dissected-out mandibles were fixed in 4% paraformaldehyde in a 0.06M Na-cacodylate buffer (pH 7.3) at 4 °C for 24 hours. Mandibles were scanned by μCT50 microcomputed tomography (micro-CT) system (Scanco Medical AG, Bassersdorf, Switzerland) at 10.0μm resolution with energy parameters of 55 KVp, 54 μA, 6W, 0.5mm AI filter, and 500 ms integration time within a field of view of 25.04 mm. DICOM files were processed by Amira software (ver. 2022.2, Thermo Fisher, https://www.thermofisher.com/us/en/home/electron-microscopy/products/​software-em-3d-vis/amira-software.html) for 3D visualization and quantification. A non-local means filter [spatial StdDev= 5, intensity StdDev = 0.2, search window [px] = 9, local neighborhood [px] = 3] and an unsharp masking [interpretation = 3D, edge size [px] = 6, edge contrast = 0.7, brightness threshold = 0] image processing filter was applied to increase the contrast between the mineralized enamel matrix and the surrounding dentin.

After adjusting the orientation of the 3D reconstituted image, enamel mineral density was measured as the average of the greyscale values of all the voxels within the semi-automatically selected enamel region on 5 frontal slices of mandibles at the selected positions. The obtained density in HU was further equated to the mineral density mg HA/cm^3^ based on the calibration parameters. On the 3D reconstitution, an enamel masking threshold of 3500 mg HA/cm^3^ was applied to segment the highly mineralizing enamel matrix above the density of dentin. The volume of the segmented enamel was measured by voxel counting. The volume was further normalized to the total mandible volume of each sample as a %.

Relative enamel density and volume between the miR-exon4 inhibitor injected group vs. controls were statistically analyzed by an independent Student’s t-test following the F-test using GraphPad Prism software (GraphPad Software). P<0.05 was considered significantly different.

### Histology on undecalcified sections

The dissected-out maxillae were fixed in 4% paraformaldehyde in a 0.06M Na-cacodylate buffer (pH 7.3) at 4 °C for 24 hours. Samples were then dehydrated with acetone and embedded in Technovit 8100 (Kulzer, Hanau, Germany) according to the manufacturer’s instructions. 2-µm-thick sections were cut and stained with Masson’s Trichrome staining and von Kossa staining, followed by Toluidine Blue staining.

### Immunohistochemistry

The 4% paraformaldehyde-fixed maxillae were decalcified in 8% EDTA (pH 7.3) and processed for routine paraffin embedding and sectioning^8^. After deparaffinization, sections underwent antigen retrieval in a citric acid-based antigen unmasking solution (Vector Laboratories Inc., Burlingame, CA) at 95 °C. They were then blocked with 5% normal swine serum for 1 hour at room temperature, followed by incubation with primary antibodies, rabbit anti-mouse RUNX2 antibody (catalog # 142160, United States Biological, Salem, MA), polyclonal rabbit anti-amelogenin long-form antibody (catalog # PA5-114845, Thermo Fisher), and rabbit anti-amelogenin exon4 antibody^15^, at 4 °C overnight. After incubation with swine anti-rabbit IgG F(ab’)2 fraction secondary antibody (Agilent Technologies, Santa Clara, CA) for one hour at room temperature, the sections treated with anti-amelogenin antibodies were incubated with ALPase-conjugated streptavidin (Vector Laboratories Inc., Burlingame, CA) for 30 minutes at room temperature. The immunoreactions were detected with the Vector® Red Alkaline Phosphatase Substrate Kit (Vector Laboratories). Levamisole (1 mM) was added to the reagents to inhibit endogenous tissue nonspecific ALPase. Counterstaining was done with methyl green. For sectioned treated with anti-RUNX2 antibody, Qdot 488 conjugated streptavidin (Thermo Fisher) was used after the secondary antibody. Hoechst 33342 was used for nuclear staining. Normal rabbit or mouse serum IgG was used as a negative control. Immunoreactions were observed under a light microscope (Nikon E800 system, Nikon TMS, Melville, NY, USA) and photographed using cellSens software (ver. 2.3, EVIDENT, Tokyo, Japan, https://evidentscientific.com/en/products/software/cellsens) to evaluate staining localization and intensity. Adobe Photoshop software (ver. 24, Adobe Systems Inc., San Jose, CA, https://www.adobe.com/products/photoshop.html) minimally adjusted image contrast.

### Cell culture with amelogenin minigene for qPCR and RIP-dPCR analysis

Amelogenin (*Amelx*) minigene vector plasmid (gifted by Dr. Michael Paine, University of Southern California) contains 5.75 kbp mouse amelogenin genomic DNA sequences from exon2 start codon to exon7 stop codon, generating alternative splice variant and miR-exon4 in LS8 and HEK293 cells^8,14,41^. QuikChange Lightning Site-Directed Mutagenesis Kit (Agilent Technologies) was used to introduce c.144+22C>A mutation.

HEK293T/17 cells (catalog # CCLZR076, UCSF Cell Culture Core Facility) were plated at a cell density of 67,000 cells/cm^2^ and cultured with DMEM + GlutaMAX (Thermo Fisher) supplemented with 10% fetal bovine serum and 1% penicillin-streptomycin. After 24 hours, minigene plasmid DNA was transfected into the cells with Lipofectamine 2000 (Thermo Fisher). After 72 hours, cells were harvested, and total RNA was extracted using the Direct-zol RNA miniprep plus kit (Zymo Research, Irvine, CA). Cytoplasmic and nuclear RNA were extracted using the Cytoplasmic and Nuclear RNA Purification Kit (Norgen Biotek Corp., Thorold, Canada). miRNA was selectively transcribed into cDNA using miRCURY LNA RT kit (Qiagen), and mRNA was reverse transcribed into cDNA using Superscript IV VILO First-Strand Synthesis Master Mix (Thermo Fisher). Expression of mature miRNA was characterized by qPCR with the miRCURY LNA SYBR Green PCR Kit (Qiagen) and a custom-made primer for miR-exon4 (Qiagen), using QuantStudio™ 6 Pro (Thermo Fisher). Expression of *Amelx exon4*, total *Amelx, SRSF2, SRSF3, SRSF6, and TRA2B* was examined by qPCR with TB Green Premix Ex Taq II (Takara Bio, Shiga, Japan) using primer sets of *Amelx exon4-6d, Amelx exon2*-*PA*28956, *SRSF2*, *SRSF3, SRSF6, and TRA2b* (Appendix Table 1). *MRPL19* was used as a reference gene for qPCR detecting mRNAs. A predesigned primer for *SNORD44* (Qiagen) was used as a reference gene for qPCR detecting cytoplasmic and total miRNA. *MALAT1* was used as a reference gene for nuclear miRNA. The Cq value of each qPCR amplification was obtained by Design and Analysis software (ver. 2.7.0, Thermo Fisher, https://www.thermofisher.com/us/en/home/technical-resources/software-downloads/​quantstudio-6-7-pro-real-time-pcr-system.html). The relative expression levels of target genes were analyzed using the ΔΔCt method^42^. The expression of each gene was calculated as a relative expression level (fold change) compared with WT.

miRNA-binding nuclear RNA was extracted using RNA immunoprecipitation (RIP) with the miRNA Target IP kit (Active Motif, Carlsbad, CA), following a modified protocol for the sample input. Instead of the total cell input, the nuclear pellet obtained by the Cytoplasmic and Nuclear RNA Purification Kit (Norgen Biotek Corp) was used as the input for the RIP procedure. miRNA-binding RNA was immunoprecipitated using an anti-Ago1/2/3 antibody or a negative control IgG provided in the kit. The extracted RNA was transcribed into cDNA using Superscript IV VILO First-Strand Synthesis Master Mix (Thermo Fisher). The amount of the *Amelx* pre-mRNA was determined as copy number per input cDNA by digital PCR (dPCR) of *Amelx* intron5-exon6d with the QIAcuity® EG PCR Kit (Qiagen) using the QIAcuity® dPCR system (Qiagen). The copy number per input cDNA of the negative-control IgG was used for normalization.

For both qPCR and dPCR, the fold change or normalized copy number/cDNA input was further normalized by the minigene transfection efficiency rate determined by the *Amelx* minigene-specific qPCR, amplifying cytoplasmic mRNA between exon2 and position PA28956 downstream of the amelogenin coding sequence but before the polyA tail^41^. The significance of differences between WT and c.144+22C>A mutation group was determined by the independent Student’s t-tests following the F-test using GraphPad Prism software (GraphPad Software). p<0.05 was considered significantly different.

### Reporter assay

HEK293T/17 cells (UCSF Cell and Genome Engineering Core) were plated at an initial density of 156,250 cells/cm^2^ and cultured with DMEM with GlutaMAX™ supplemented with 10% FBS and 1% P/S for 24 hours at 37 ℃ with 5% CO_2_. A dual reporter vector encoding SV40 promoter-driven secreted Gaussia luciferase (Gluc) followed by mouse *Srsf6* 3'UTR and CMV promoter-driven secreted Alkaline Phosphatase (SEAP) (GeneCopoeia, Rockville, MD) or miRNA Target clone negative control vector (n=4) (GeneCopoeia) was co-transfected with either mirVana miR-exon4 mimic oligonucleotide (n=4) (ACUGACAGGACUGCAUUA, Thermo Fisher) using lipofectamine^TM^ 2000 (Thermo Fisher). After 72 hours of culture at 37 ℃, the secreted Gluc and SEAP in the culture medium were measured using Secrete-Pair™ Dual Luminescence Assay Kit (GeneCopoeia) and SpectraMax iD3 multi-mode microplate reader (Molecular Devices, San Jose, CA). The measured luciferase activity of each sample was normalized by SEAP, an internal control for transfection. The difference in the signal between with and without miR-exon4 was analyzed for each vector by the Kolmogorov-Smirnov test. For both analyses, p<0.05 was considered significantly different.

### Enhanced crosslinking and immunoprecipitation (eCLIP)-seq

The eCLIP-seq workflow was adapted from Blue et al.^44^, with modifications tailored to this study. HEK-293 cells (~10 million) were transfected with the WT and c.144+22C>A mutant *Amelx* minigene vector plasmid. Once expanded, the cells were harvested and UV-crosslinked at 400 mJ/cm^2^ for 1 cycle and 200 mJ/cm^2^ for 2 cycles using a Stratalinker. Cells were then lysed and treated with Turbo DNAse and RNAse I (30 units, 5 minutes at 37°C). 2% of the WT and Mutant lysates were set aside as their respective Input samples, and the rest were subjected to immunoprecipitation (IP). Overnight IP was performed using magnetic beads pre-bound to Ago1/2/3 antibody (catalog # 39937, Active Motif, Carlsbad, CA), after which the pulled-down fractions and reserved Input samples were ligated to a 3’ RNA linker. IP fractions were then eluted from beads and resolved alongside the Input samples on duplicate SDS-PAGE gels, each transferred to a nitrocellulose and a PVDF membrane. Following standard immunoblotting procedures, the PVDF membrane was probed with antibodies against Ago1/2/3 proteins. The developed bands were used as a guide to excise the corresponding Ago1/2/3 region on a nitrocellulose membrane, along with a 75 kDa area above it. The excised membrane was extracted for RNA and reverse-transcribed to cDNA, after which 3’ DNA adapter ligation was performed. Library amplification was performed using PCR cycles determined by qPCR analysis of each sample. Libraries were then size-selected by gel extraction and analyzed on the Tape Station for quality check. Prepared libraries were sequenced on the Illumina NovaSeq X Plus at Novogene (Sacramento, CA).

Raw eCLIP sequencing data were processed using a custom UNIX pipeline implemented in the eclip_env conda environment. Sequencing adapters were first trimmed from raw FASTQ files using Cutadapt (v4.4) with parameters -a AGATCGGAAGAGCACACGTCTGAACTCCAGTCA -m 18 -O 5 --match-read-wildcards, retaining reads ≥18 nt. Trimmed reads were then processed with UMI-tools (v1.1.5) to extract 8-nt unique molecular identifiers (UMIs) from the 5′ end of each read (--bc-pattern=NNNNNNNN) and to remove PCR duplicates after alignment.

Reads were aligned to the Gencode M30 (GRCm39 and GRCh38) reference genome using STAR (v2.7.10a) with the following key parameters: --outFilterMultimapNmax 10, --alignSJoverhangMin 8, --alignSJDBoverhangMin 1, --outFilterMismatchNoverLmax 0.1, --alignIntronMax 1,000,000, and --alignMatesGapMax 1,000,000. Alignments were output as coordinate-sorted BAM files and indexed using samtools (v1.17). PCR duplicates were removed using umi_tools dedup with --extract-umi-method=read_id.

Peak calling was performed with a custom region-specific filtering strategy to directly quantify reads overlapping the predicted 18-nt binding region plus 2 nt within intron4 (chrX:167965539–167965556). Using a Python script built with pysam (v0.22.0), all deduplicated BAM files were scanned to extract reads that either (i) started or ended within this 18+2-nt window, or (ii) spanned the region with at least one mismatch (based on the *MD* tag, representing crosslink-induced mutations). Reads meeting these criteria were written to new BAM and TSV files for both WT and c.144+22C>A samples, along with associated metadata (chromosome, start, end, strand, and mismatch count). The filtering was performed independently for each library, and output files were indexed using Samtools for visualization in IGV. The peak call numbers were normalized to the *Amelx* minigene reads. The number of reads mapped to the *Amelx* minigene was normalized to the total unique reads and further adjusted using the transfection rate (WT/c.144+22C>A = 1/0.796), based on the INPUT data mapped to mouse *Amelx*.

## Supplementary Information

Supplementary Information.

## Data Availability

The datasets generated during and/or analyzed during the current study are available from the corresponding author on reasonable request.
