# Supplementary material for: A splicing-derived microRNA from amelogenin exon4 regulates enamel formation via control of exon4 splicing and amelogenin expression
Source: Sci Rep. 2026 Feb 25;16:11044. doi: 10.1038/s41598-026-40706-0 (PMC13043915; doi:10.1038/s41598-026-40706-0)
Supplement: Supplementary file 1 — Supplementary Information. [file 41598_2026_40706_MOESM1_ESM.pdf]

Appendix Table 1: Primer sequences

Gene		Sequence (5'-3')
Nfia	F	AAGCCTCCAACCACATCAAC
	R	TTTACAAAGCTTGGATCCC
Prkch	F	TCCGGCACGATGAAGTTCAAT
	R	TACGCTCACCGTCAGGTAGG
Runx2	F	CGGCCCTCCCTGAACTCT
	R	TGCCTGCCTGGGATCTGTA
Amelx exon4	F	AAGTCACATTCTCAGGCTATCAATACT
Amelx intron5	F	GAGCCAATGGTAAACCTGACTCTTT
Amelx exon2	F	GTTTGCCTGCCTCCTGGGAGCAGC
*PA*28956	R	CAACTAGAAGGCACAGTCGAGGCTGATCA
Amelx exon6d	R	ACTTCTTCCCGCTTGGTCTTGTCT
Srsf2	F	CAGAAGAAGAGGGAGCAGTTT
	R	GACCGATGGACTGAGTTTGT
Srsf3	F	AGTGTGTGGGTTGCTCGAAA
	R	GTTCTTCCATCTAGTTCCCGGA
Srsf6	F	ACATAGGACGCCTGAGCTACA
	R	CCGTACCCATTTTTGAGGTCG
Tra2b	F	AATCCCGTTCTGCTTCCCG
	R	TCGTGACCTTGTATAATGCCTTC
SRSF2	F	CCCGATGTGGAGGGTATGAC
	R	GAGACTTCGAGCGGCTGTAG
Mrpl19	F	ACGGCTTGCTGCCTTCGCAT
	R	AGGAACCTTCTCTCGTCTTCCGGG
MRPL19	F	GGAATGTTATCGAAGGACAAGGT
	R	ATCCTCGGGTCCAGGAGATTCAGG
MALAT1	F	TTGGTGAATTGATAAGTAAAGGCAG
	R	TTTTTGGCATATGCAGATAATGTTC

Appendix Figure 1

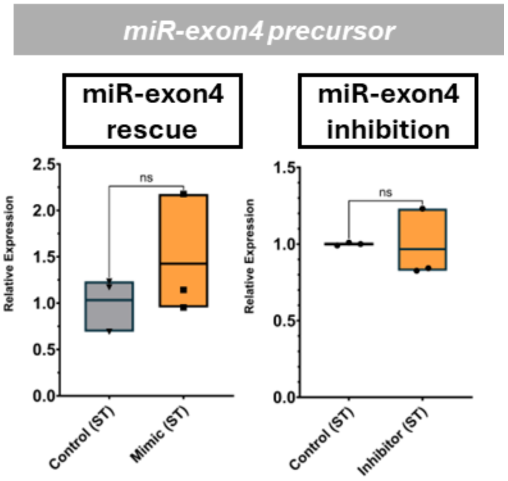

qPCR analysis of miR-exon4 precursor: Total RNA extract of enamel organs from miR-exon4 mimic injection, miR-exon4 inhibitor injection, and respective control group were reverse transcribed using miScript RT kit (Qiagen). The miR-exon4 precursor was amplified by qPCR with a primer set of F: CTATCAATACTGACAGGAC and R: GCAGTATGAAATATAGACTCAC. A predesigned primer for *Snord95* (Qiagen) was used for a reference gene. The data analysis follows the method in the main text. In both miR-exon4 mimic injection to *Amelx* KO mice (miR-exon4 rescue) and miR-exon4 inhibition on Amelx WT mice, miR-exon4 precursor reflecting the endogenous miR-exon4 biosynthesis did not significantly differ from controls. Each box represents the minimum to maximum data collected (shown as individual dots), with the middle line at the mean. n=3/each group

Appendix Figure 2

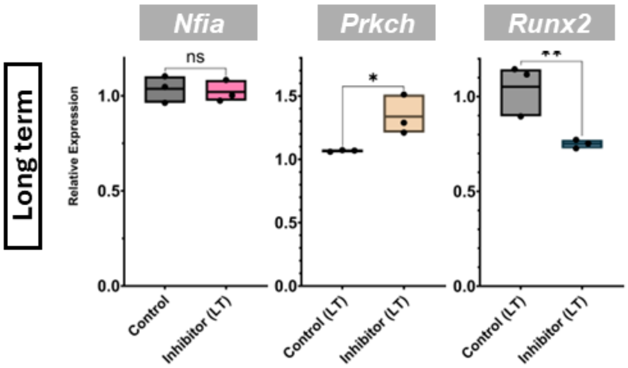

qPCR analysis of miR-exon4 targets after long-term miR-exon4 inhibition: While miR-exon4 inhibition still causes significant upregulation of *Prkch* and downregulation of *Runx2*, *Nfia* does not respond to the miR-exon4 inhibition after long-term treatment. Each box represents the minimum to maximum data collected (shown as individual dots), with the middle line at the mean. n=3/each group *: $p<0.05$ and **: $p<0.01$

Appendix Figure 3

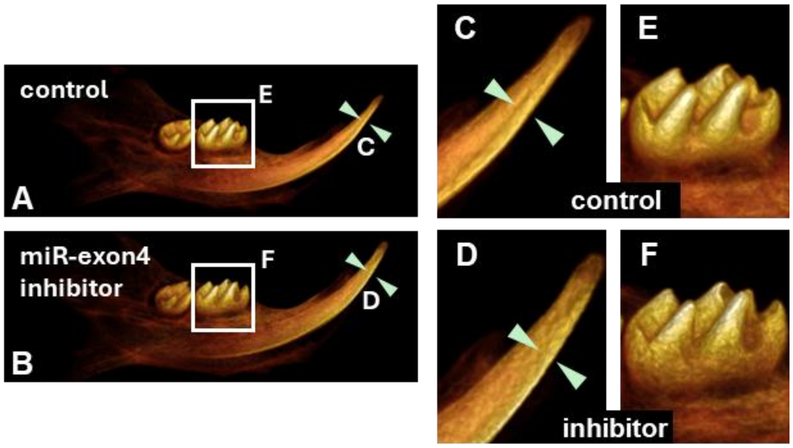

The 3D tomography from the micro-CT scan shows the incisors and molars in images A (control) and B (miR-exon4 inhibitor injection). In the incisor (indicated by D), the border between the enamel and the root appears vague in the inhibitor-injected group compared to the control group (shown in C). Additionally, the molars in the miR-exon4 inhibitor injected group exhibit rough surface mineralization (F) compared to the control group (E). Overall, these findings indicate that the inhibitor-injected group has lower levels of mineralization than the control group.

Appendix Figure 4

A

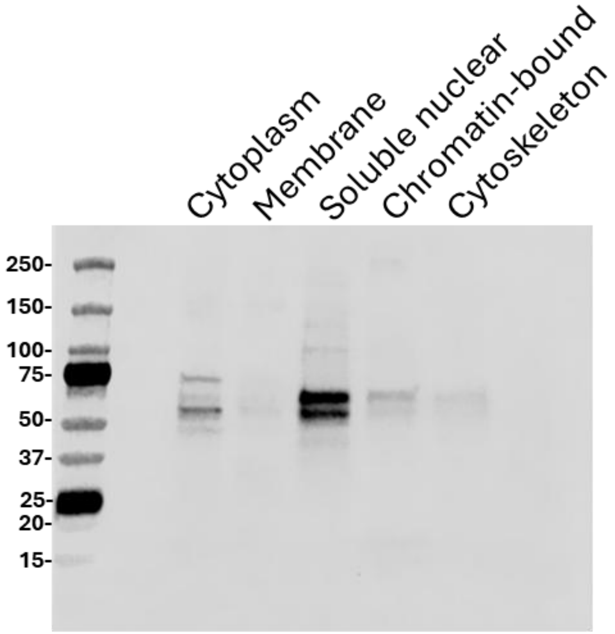

B

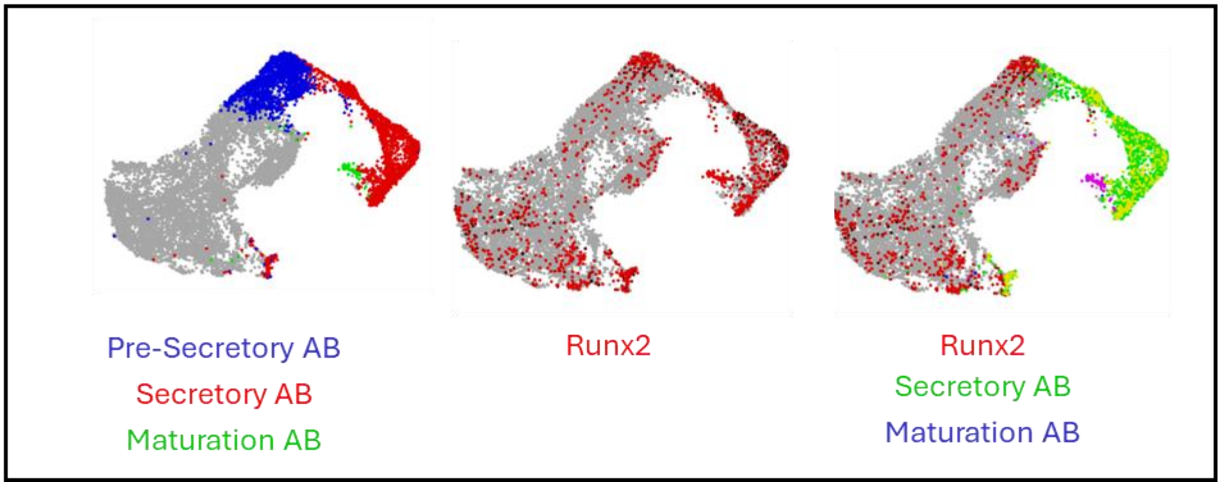

Runx2 localization in secretory ameloblasts. (A) Western blotting of subcellular fractions from LS8 ameloblastic cells shows Runx2 in the cytoplasm and nuclear fractions, with faint signals in the chromatin-bound and cytoskeleton fractions. LS8 cells were plated at 70,000 cells/cm^2 and cultured with DMEM + GlutaMAX (Thermo Fisher Scientific) supplemented with 10% fetal bovine serum and 1% penicillin-streptomycin. After 2 days of culture, cells were harvested using the Subcellular Protein Fractionation Kit (Thermo Fisher Scientific). 15 mg of protein from each fraction was separated on 4-15% gradient acrylamide gels under reducing conditions and transferred onto PVDF membranes. Membranes were blocked with Odyssey blocking buffer (LI-COR Biosciences, Lincoln, NE) for one hour at room temperature, followed by overnight incubation with rabbit anti-RUNX2 antibody (catalog #NBP2-67777, Novus Biologicals, Centennial, CO). IRDye® 680-conjugated Donkey anti-rabbit antibody was used as the secondary antibody for a one-hour incubation at room temperature. Bands were visualized by Odyssey® CLx (LI-COR Biosciences) scanner, and band signal was analyzed using Empiria software (LI-COR Biosciences). (B) Using an integrated transcriptome atlas based on the 9 published scRNA sequencing datasets of the mouse tooth in .loom format and the Scope platform (Hermans et al. 2022) mRNA expression of Runx2 is clearly detected in the secretory stage of ameloblasts, in addition to the maturation stage.

Appendix Figure 5

SR protein	Score in mouse	Score in human	Prediction of binding to Amelx exons 4 or 5
SRSF2	0.637	N/A	Exon 4
SRSF3	0.406	0.530	Exons 4 and 5
SRSF6	N/A	0.387	Exons 4 and 5
SRSF7	0.423	0.867	N/A
SRSF8	N/A	0.397	N/A
SRSF9	0.386	N/A	N/A
SRSF10	0.523	0.366	N/A
SRSF11	0.386	N/A	N/A
SRSF12	0.444	N/A	N/A
TRA2B	N/A	0.523	Exon 5

SR-proteins predicted as the direct target of miR-exon4 and their potential binding to exons 4 and 5. Please refer to the main text for the algorithms used for the prediction. Higher prediction scores correspond to a higher possibility of correct prediction.

Appendix Figure 6

```
Version: RNAhybrid 2.2

searching
dataset: 1
mde of Ms_miR-exon4: -37.799999
Individual hits
```

```
dataset: 1
Target: Ms_Amelx_int3-ex4-int4-ex5
length: 1415
MiRNA: Ms_miR-exon4
length: 18

mfe: -18.3 kcal/mol
p-value: 1.000000e+00

Position: 1341
target 5' A          UAA     U   3'
           AAUGCA UCC   UGUC
           UUACGU AGG   ACAG
miRNA  3' A      C          UCA 5'
```

chrX:11298145

intron4

GTGAGTCTATATTTCATACTGCATTAGTGAGTTTAGTATTTAGTTCATA
TGTAATTAAACTGAATCAAATGCATC**C**T**AA****T**GTCTCTTTCTCTTAAG

chrX:11298235

RNA hybrid prediction between Amelx intron4 and miR-exon4. The predicted binding site in the intron4 sequence is in red. The green highlight indicates the actual hybrid sequence. The red bold sequence indicates the human branch point sequence (YTNAY) predicted by Splice-Alternative Profile Predictor (SpliceAPP) utilizing the SVM BP Finder algorithm (Corvelo et al. 2010).

Appendix Figure 7

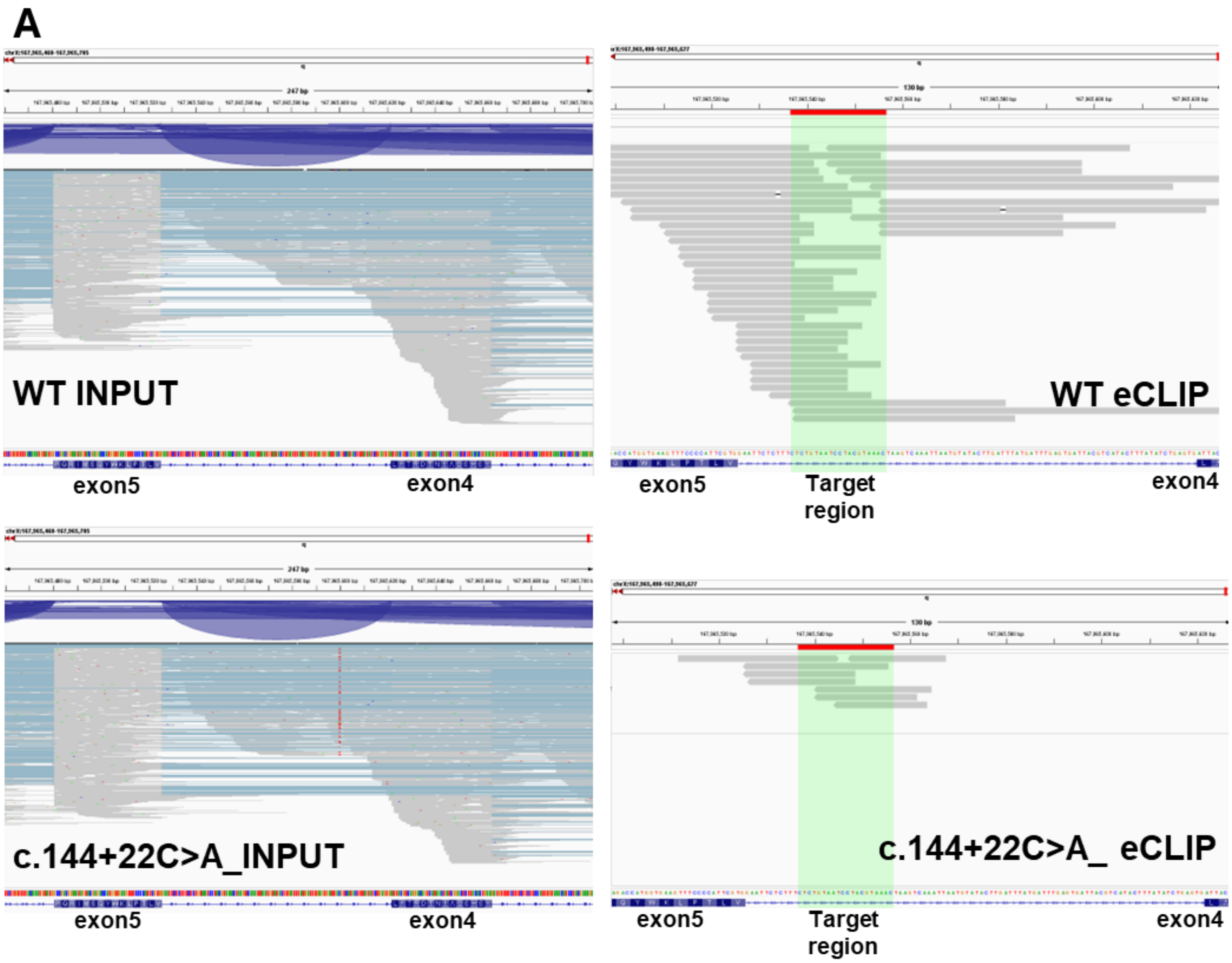

A) Visualization of reads mapped to the *Amelx* exon4-intron4-exon5 region using IGV.

References

Corvelo A, Hallegger M, Smith CW, Eyras E. 2010. Genome-wide association between branch point properties and alternative splicing. PLoS Comput Biol. 6(11):e1001016.

Hermans F, Bueds C, Hemeryck L, Lambrichts I, Bronckaers A, Vankelecom H. 2022. Establishment of inclusive single-cell transcriptome atlases from mouse and human tooth as powerful resource for dental research. Frontiers in Cell and Developmental Biology. Volume 10 - 2022.
